# Central Hypogonadotropic Hypogonadism: Genetic Complexity of a Complex Disease

**DOI:** 10.1155/2014/649154

**Published:** 2014-09-01

**Authors:** Marco Marino, Valeria Moriondo, Eleonora Vighi, Elisa Pignatti, Manuela Simoni

**Affiliations:** ^1^Unit of Endocrinology, Department of Biomedical, Metabolic and Neural Sciences, University of Modena and Reggio Emilia, NOCSAE, Via Pietro Giardini 1355, 41126 Modena, Italy; ^2^Center for Genomic Research, University of Modena and Reggio Emilia, Via Giuseppe Campi 187, 41125 Modena, Italy; ^3^Azienda USL of Modena, Via San Giovanni del Cantone 23, 41121 Modena, Italy

## Abstract

Central hypogonadotropic hypogonadism (CHH) is an emerging pathological condition frequently associated with overweight, metabolic syndrome, diabetes, and midline defects. The genetic mechanisms involve mutations in at least twenty-four genes regulating GnRH neuronal migration, secretion, and activity. So far, the mechanisms underlying CHH, both in prepubertal and in adulthood onset forms, remain unknown in most of the cases. Indeed, all detected gene variants may explain a small proportion of the affected patients (43%), indicating that other genes or epigenetic mechanisms are involved in the onset of CHH. The aim of this review is to summarize the current knowledge on genetic background of CHH, organizing the large amount of data present in the literature in a clear and concise manner, to produce a useful guide available for researchers and clinicians.

## 1. Introduction

The physiological function of the human hypothalamic-pituitary-gonadal (HPG) axis is based on the pulsatile release of hypothalamic gonadotropin-releasing hormone (GnRH) [1]. In vertebrates, the decapeptide GnRH regulates the secretion of luteinizing hormone (LH) and follicle-stimulating hormone (FSH) from anterior pituitary gonadotropes, governing the onset of puberty, gametogenesis, and in females, estrous/menstrual cycling [2].

During postnatal life, GnRH-secreting neurons are integral members of the HPG axis. During embryonic development, these cells originate from an extracerebral region, namely, the nasal placode, and migrate to the hypothalamus, closed to olfactory-vomeronasal nerves (VNNs) [2, 3]. The correct development and coordinated function of the GnRH-secreting neurons and the gonadotropes are essential for the correct activation of the gonads during foetal life and the neonatal period (the so-called “minipuberty”). After a dormant phase during infancy and childhood, HPG activity is resumed at the time of the puberty and throughout the adult reproductive age [1].

In human males, after birth, the HPG axis is active until approximately 6 months of life, with gonadotropin and sex steroids concentrations peaking between 4 and 12 weeks of life. After this period, GnRH pulsatility declines and the HPG axis becomes quiescent throughout childhood. Onset of puberty is characterized by pulsatile GnRH release from the hypothalamus that stimulates pituitary LH secretion, which, in turn, drives testosterone production by testicular Leydig cells [4, 5]. GnRH also increases FSH secretion, which promotes maturation of the seminiferous tubules and spermatogonia [5]. Adrenarche, at about 6-7 years of age, starts when the adrenal androgen, DHEA, DHEAS, and androstenedione concentrations, produced by adrenal zona reticularis, begin to increase in the circulation [5, 6]. Adrenarche continues up to approximately 14 years of age in both sexes [4, 7]. Pubarche frequently starts simultaneously with testicular development, while axillary hair growth occurs at about the time of peak height velocity (about 14 years of age) [8]. Delayed puberty is usually defined as the absence of signs of puberty by age 14 in boys [7].

In human females, during childhood the ovarian cycle, controlled by HPG axis, is quiescent [9]. Adrenarche (6–8 years of age), clinically evident with pubic and axillary hair growth [7, 9], is followed by gonadarche and during this period the nocturnal LH pulse occurs, leading to ovarian production of testosterone and progesterone. Gonadal steroids facilitate the pubertal growth spurt and breast development (thelarche), that generally begins about age 8-9 years, leading to menarche [7, 9]. A growth spurt occurs at 12 years of age, and subsequently, the first menstrual cycle occurs (menarche) [7]. Menarche, induced by follicle growth and estradiol production, occurs with the initial shedding of the endometrium with subsequent bleeding. Female puberty ends when women ovulate and repeated ovulatory cycles ensure reproductive competence [9, 10].

The complex disease called central hypogonadotropic hypogonadism (CHH) is able to seriously compromise the physiologic function of the HPG axis in both sexes. CHH is characterized by delayed or absent sexual development and infertility associated with inappropriately low gonadotropins (LH and FSH) and sex steroids (testosterone or estradiol) levels in the absence of anatomical or functional abnormalities of the HPG axis [11–13]. In patients with normal levels of circulating gonadotropins, LH and FSH are secreted in a nonpulsatile manner and are ineffective at the target level [1]. CHH is a rare disease with an incidence of 1 : 8000 females and 1 : 4000 males [1]. The disease associated with a normal sense of smell, occurring in 40–45% of CHH patients [13], is called normosmic Idiopathic Central hypogonadism (nICH) or normosmic hypogonadotropic hypogonadism (nHH). When a defective sense of smell, hyposmia or anosmia, is associated with hypogonadotropic hypogonadism, in 60% of patients [13], the disease is called Kallmann syndrome (KS), explained by the common embryonic origins and developmental pathways of GnRH and olfactory neurons [1, 11, 12, 14]. CHH is either congenital or acquired, and it can be isolated or combined with other pituitary hormone defects [1]. Male patients affected by CHH frequently present a defective androgenisation and growth at a peripubertal age, but micropenis and cryptorchidism may already be evident in the neonatal period, indicating a defective activation of HPG during prenatal development. Female patients generally show primary amenorrhea and growth retardation. Midline and/or kidney defects may be present and can be linked to specific modes of inheritance [1, 15].

In the past two decades, hypogonadotropic hypogonadism was considered an irreversible disease, to treat with a long-life hormonal exposure. By contrast, it is well known that a small proportions of male patients, up to 10%, after exposure to androgens therapy, may undergo reversal of hypogonadism [16]. Patients affected by KS or normosmic HH with mutations in fibroblast growth factor receptor 1 (*FGFR1*),* KAL1*,* GNRHR*, and* CHD7* genes or with still unknown genetic defects were reported to present a reversible phenotype, after therapy withdrawal [17–21]. In these patients a spontaneous recovery of LH pulsatile secretion occurred together with normalization of the testosterone level after therapy suspension [20]. Although the precise mechanism of reversal of hypogonadotropic hypogonadism is unclear, plasticity of the GnRH-producing neurons in adulthood could be involved [20]. The ability of the nervous system to adapt in response to environment is a striking feature of the vertebrate brain. Although, neurogenesis in humans occurs primarily during embryonic and early postnatal stages, multipotential progenitor cells in the subcortical white matter of the adult human brain have been identified as having the potential to replace neuronal lineages [22]. Furthermore, the neurons in the olfactory epithelium and in the dentate gyrus of the hippocampus are generated throughout life [23–25] and their generation appears to be modulated by sex steroids [26]. A current hypothesis to explain reversal of hypogonadotropic hypogonadism suggests the action of sex steroids in enhancing the plasticity of the neuronal network producing GnRH in the adult human brain [20].

## 2. Diagnosis and Treatment of CHH

The diagnosis may be suspected before puberty when boys present micropenis and/or unilateral or bilateral cryptorchidism and in the presence of other associated congenital abnormalities, such as midline defects (cleft palate, short metacarpals, hearing loss, bimanual synkinesia, etc.) [1, 27]. In contrast, newborn girls have no obvious abnormal characteristics suggesting a congenital CHH diagnosis. Adult-onset CHH is characterized by secondary amenorrhea, decreased libido, infertility, and osteoporosis, in women and symptoms of decreased libido, lack of morning erection, erectile dysfunction, inability to perform vigorous activity, depression, fatigue and infertility, in men [28].

The measurement of morning total testosterone is strongly recommended as the initial diagnosis test [29], especially assessing free or bioavailable testosterone levels [30]. Although widely used in the past, the practical value of the GnRH test has been questioned because this test does not provide extra diagnostic information compared to baseline gonadotropin levels [28]. The pituitary function can be first evaluated by basal hormonal levels (measured by ultrasensitive assays). Thyroid function should be assessed by TSH combined with free T4. IGF-I can be used to evaluate the somatotropic axis, whereas secondary adrenal deficiency can be assessed by measuring a morning cortisol and ACTH. Anosmia can be diagnosed by questioning the patient and by olfactometry as the University of Pennsylvania smell identification test, useful to determine a normal or partially defective olfaction [28].

Magnetic resonance imaging (MRI) of the hypothalamus-pituitary region is very useful in the management of CHH because MRI can demonstrate a malformation or tumors. Renal ultrasound examination is usually recommended to patients with syndromic CHH, such as Kallmann syndrome. The genetic study is usually the final step in the CHH investigation and a complete clinical characterization could be very useful in the selection of the gene/s to be screened [28].

The aims of therapy in hypogonadal adolescents or young adults are the induction and maintenance of normal puberty and induction of fertility.

Testosterone therapy is recommended in adult men with symptomatic androgen deficiency to improve sexual function and to increase muscle mass and strength. Testosterone is the primary useful treatment to induce and maintain secondary sexual characteristics and sexual function in affected men, but it does not restore fertility [28]. Several testosterone formulations are currently available such as intramuscular injections of long-acting testosterone esters, gel formulations, or testosterone patches applied nightly [29]. When fertility is desired, gonadotropin therapy is necessary to induce spermatogenesis in affected males. The common gonadotropin therapy combines human chorionic gonadotropin (hCG) and follicle stimulating hormone (FSH) [31, 32].

## 3. Genetic Basis of CHH

A small percentage of patients (approximately 4%) shows a chromosomal rearrangement as cause of CHH or KS [7, 33], but the majority of hypogonadic patients harbour a mutation in a single or more genes.

Isolated GnRH deficiency, caused by defects in the secretion or action of hypothalamic GnRH, is one of the rare genetic diseases originally thought to be strictly monogenic but the numerous studies about this disorder led to the discovery of several new loci [34] (Table 1), with key roles for the developmental and neuroendocrine control of human reproduction [1, 12, 14]. So far, twenty-four genes have been identified (Table 1).

**Figure 1:**
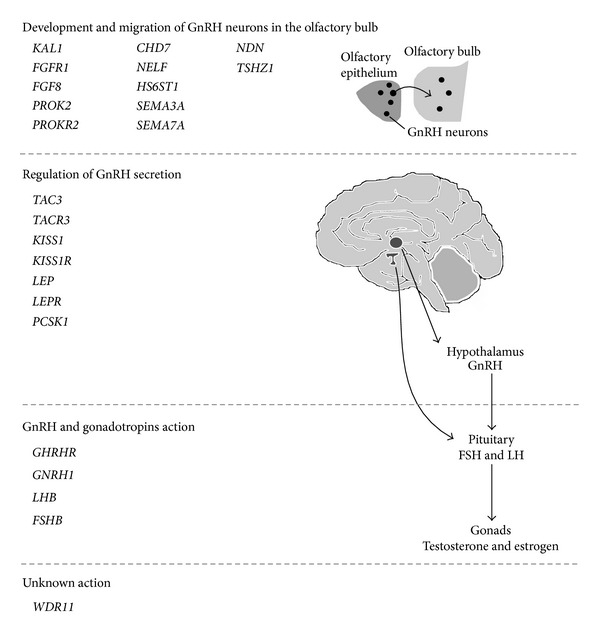
Scheme of all human CHH-related genes involved and supposed to be involved at different steps in the hypothalamic-pituitary-gonadal axis development and functioning. All three main steps leading to the HPG axis formation are reported in this picture. Upper side: list of the genes, implicated in the development of GnRH neurons and their migration towards the olfactory bulb, during the embryonic development. Middle part: genes involved in the regulation of GnRH secretion. Lower side: genes implicated in the direct action of GnRH on pituitary and in its indirect action on gonads.

The proteins, encoded by the genes involved so far in this pathology, have been grouped, according to their function, in three functional categories: development and migration of GnRH neurons, regulation of GnRH secretion, and GnRH and gonadotropins action [1, 11, 12, 35] (Figure 1). However, the function of some genes, recently identified, is not fully clear.

Although multiple mutations have been identified in each gene, in the majority of affected patients (approximately 60%), no mutations can be found, signifying that yet more disease loci remain to be discovered [14].

### 3.1. *KAL1*

The* KAL1* gene, located on the short arm of chromosome X (Xp22.3), contains 14 exons and encodes the extracellular matrix glycoprotein anosmin-1, which appears to be involved in the migration of GnRH and olfactory neurons during embryologic development [36] (Figure 1). Hypogonadism in KS, due to GnRH deficiency, probably results from a failure of the embryonic neuronal migration, and the defective sense of smell is related to the hypoplasia or aplasia of the olfactory bulbs and tracts [37]. Anosmin 1 is a secreted protein containing a cysteine-rich region, a highly conserved four disulfide core whey acid protein (WAP) domain, four fibronectin type III (FNIII) repeats (including FNIII-1, FNIII-2, FNIII-3, and FNIII-4 repeat), and a C-terminal histidine-rich region [36].

Mutations in the* KAL1* gene were first described in males with X-linked recessive KS [38]. So far, in the literature, over 60 different mutations were described including deletions, missense, frameshift [39], and gross rearrangements [37, 40]. About half of the mutations fall in WAP domain and in FNIII repeats suggesting their important role in the correct function of anosmin-1 [41]. All* KAL1* mutations account for 33 to 70% of familial cases of KS and 3.1 to 27.8% of apparently sporadic forms of known KS [36, 37].

### 3.2. *FGFR1 (KAL2) *and* FGF8 (KAL6)*

*FGFR1*, encoding fibroblast growth factor receptor-1, is a member of the receptor tyrosine kinase superfamily. FGF signalling controls cell proliferation, migration, differentiation, survival and plays essential roles in various processes of embryonic development. In the presence of heparin sulphate proteoglycans (HSPG), FGF8 (fibroblast growth factor-8) binds with high affinity to FGFR1 and induces receptor dimerisation, and its activation [42, 43]. The FGFR1 is involved in gastrulation, organ specification, patterning of many tissues, including the brain, as well as the development of the olfactory system [43] (Figure 1).

Both* FGFR1* and* FGF8*, mapping on 8p12 and 10q24, respectively, can cause an autosomal dominant form of KS. The majority of mutations in* FGFR1* or* FGF8* are missense mutations. Notably, about 30% of the FGFR1 variants found are de novo mutations [39]. In KS patients, skeletal anomalies such as syndactyly, polydactyly, or camptodactyly were exclusively seen in presence of* FGFR1*/*FGF8* mutations [44].

### 3.3. *PROK2 (KAL4) *and* PROKR2 (KAL3)*

The* PROK2* gene, on chromosome 3p13, encodes the protein prokinetecin 2, an 81-amino acid peptide that signals via the G protein-coupled product of the* PROKR2* gene, on chromosome 20p12.3 [45]. The amino-terminal domain of prokineticin 2 contains a sequence of six amino acid residues (AVITGA), which is conserved in all mammalian and nonmammalian orthologs. Prokineticins can bind to two different G protein-coupled receptors,* PROKR1* and* PROKR2*, sharing about 85% sequence identity. They have a central core, formed by seven transmembrane domains (TM1–TM7), connected by intracellular and extracellular loops.* PROKR1* is mainly expressed in peripheral tissues, including endocrine glands whereas* PROKR2* shows relatively localized distribution in the central nervous system, in particular in the subventricular zone (SVZ) and in the olfactory bulbs [46]. It has been shown that this ligand-receptor system is essential for normal olfactory bulb and reproductive system development in mice and in patients with CHH. Homozygous mutant* Prokr2* knockout mice show abnormal development of the olfactory bulb and severe atrophy of the reproductive system, but no significant abnormalities were observed in the heterozygous mice [37]. The knockout models for either ligand (*Prok2*) or receptor (*Prokr2*), revealed a role in olfactory bulb morphogenesis and sexual maturation, indicating* PROK2* and* PROKR2* as strong candidate genes for human GnRH deficiency [47]. Mutations described within the* PROK2*/*PROKR2* system account for less than 10% of subjects with KS and normosmic HH [37]. Nonreproductive, non-olfactory clinical anomalies associated with Kallmann syndrome seem to be restricted to patients with monoallelic mutations [45].

### 3.4. *CHD7 (KAL5)*

The chromodomain helicase DNA binding protein 7 (*CHD7*), on chromosome 8, encodes a chromatin-remodelling factor belonging to a family of nine CHD proteins and having in common the ability to utilize ATP hydrolysis to alter nucleosome structure [45, 48]. The large 2997 amino acid CHD7 protein contains two important chromodomains at its N terminus [48]. Chromodomains have been thought to mediate chromatin interactions and were found to interact with DNA, RNA, and histone targets [48].

Heterozygous mutations in* CHD7* are found in more than 60% of the patients with typical CHARGE syndrome, a multisystem autosomal-dominant or sporadic disorder including coloboma, heart anomalies, choanal atresia, retardation, genital, and ear anomalies [49]. In the literature 3 to 5% of patients with CHH or KS were found to have a* CHD7* mutation [50].

Previous studies on* CHD7* suggested that the analysis of this gene should be performed in KS patients having at least two CHARGE syndrome features. Following this guideline, Bergman and colleagues [51] performed the* CHD7 *analysis in a cohort of 36 Dutch KS patients (previously excluded to carry mutations in FGFR1, PROK2, PROKR2, and FGF8), identifying 3 heterozygous* CHD7* mutations in patients having same features of CHARGE syndrome. Conversely, in the study of Kim et al. [48], mutations in this gene were found also in KS patients not carrying any CHARGE features. Thus, considering these findings, new studies on* CHD7* and hypogonadic patients are needed.

### 3.5. *NELF*

NMDA receptor synaptonuclear signalling and neuronal migration factor, also known as Nasal Embryonic LHRH Factor (*NELF*), maps on chromosome 9. The mode of inheritance of CHH is likely to be autosomal recessive because biallelic mutations, reducing protein expression in vitro, have been described in the literature in only one KS patient without mutations in other genes, whereas heterozygous* NELF* mutations were only found in affected normosmic HH/KS patients with heterozygous mutations in another gene [52].

*NELF* is a good candidate gene for a role in GnRH neuron migration, mammalian puberty, and the pathophysiology of KS. Over ten years ago, the mouse NELF was cloned from a differential cDNA library screen of migratory versus nonmigratory GnRH neurons [53]. Its expression was found to be aligned along the plasma membrane of olfactory and GnRH neurons before they enter the hypothalamus and is downregulated when GnRH neurons reach the forebrain. Moreover, reducing NELF protein expression, GnRH neurons are reduced in number and GnRH nerve fiber decrease in complexity and length [53]. Two studies implicated* NELF* in KS [54, 55]. Subsequently, that data were confirmed by Xu et al. [56], who correlated human* NELF* mutations with monogenic normosmic HH/KS. Xu and colleagues [56] demonstrated that NELF protein was more expressed in migratory versus postmigratory GnRH neuronal cells and NELF knockdown dramatically impairs GnRH neuronal cell migration in vitro. A functional nuclear localization signal and two putative zinc fingers identify NELF as a nuclear protein, possibly a transcription factor [56, 57].

### 3.6. *GNRH1 *and* GNRHR*

GnRH plays a key role in the control of reproductive function (Figure 1). It is synthesized in a small number of hypothalamic neurons and released in a pulsatile manner into the hypophyseal portal circulation reaching the anterior pituitary, where, binding to specific receptors (GnRHRs), regulates gonadal steroidogenesis in both sexes, stimulating synthesis and release of the two gonadotropins (LH and FSH) [58] (Figure 1). Human* GNRH1*, on chromosome 8, composed by four exons separated by three introns, encodes a 92 amino acids preproprotein (pre-pro-GnRH), consisting of a signal sequence (23 amino acids) sequentially followed by two serine residues, the GnRH decapeptide, a GKR sequence, and a 56-amino acid peptide called GAP (GnRH-associated peptide) [58, 59]. The mature decapeptide sequence is conserved among most mammals [60].

In two studies, mice, carrying a deletion of* Gnrh1*, showed a complete absence of GnRH synthesis [61, 62]. Moreover, these mice were sexually immature, infertile, and exhibited low sex steroid and gonadotropin levels [61, 62]. These findings in the mouse suggested that mutations in human* GNRH1* could cause CHH. Variants of the human* GNRH1* gene are very rare. Naturally occurring mutations in human* GNRH2* were not described so far. The p.R31C mutation is the sole missense mutation affecting the mature GnRH decapeptide sequence, encoded by* GNRH1*, described so far [60]. This mutation, in which arginine is substituted by cysteine in position 8 of the mature decapeptide, represents a hot spot [52, 59].

The other variants described were detected in the extra decapeptide positions such as p.R73X, p.T58S, and p.V18M [59]. All these variants were described in heterozygous state but curiously, Chan et al. [59] reported also a homozygous mutation, p.G29GfsX12, in a male patient with severe congenital CHH. This single base-pair deletion causes a frameshift that was predicted to disrupt the GnRH decapeptide [59].

The GnRH receptor (*GNRHR*) gene, differently from that encoding its ligand, accounts for many inactivating mutations, resulting in impairment of GnRH action [63]. GNRHR is a 328-amino acid protein encoded by a gene located on chromosome 4q21.2. Its activation results in increased activity of phospholipase C and mobilisation of intracellular calcium [1]. GNRHR contains seven transmembrane domains and an extracellular 35-amino acid amino-terminal domain with two putative glycosylation sites. Interestingly, this receptor does not have a carboxy-terminal cytoplasmic tail, thus, it internalizes relatively slowly and it does not rapidly desensitize [58]. Inactivating mutations of* GNRHR* were the first to be recognized as monogenic causes of CHH condition [64]. Null* GNRHR* mice models display a similar phenotype to human CHH [1]. Over 22 human* GNRHR* inactivating mutations, with no hotspot, were described [63] and these different genotypes result in a wide phenotypic spectrum, ranging from fertile eunuch syndrome and partial hypogonadotropic hypogonadism to the most complete form of GnRH resistance, characterized by cryptorchidism, micropenis, undetectable gonadotropins and the absence of pubertal development [1]. Although many defects in a large number of different genes were associated to CHH,* GNRHR* is still the most commonly affected gene in this pathogenic condition [64]. Since the vast majority of patients harbouring* GNRHR* mutations are resistant to GnRH, the effective fertility treatment is achieved with gonadotropins [63].

### 3.7. *LEP *and* LEPR*

Adipose tissue, through expression and secretion of leptin, plays a dynamic role in whole-body energy homeostasis by acting as an endocrine organ [65]. Twenty years ago, a mouse model, named “*ob/ob,*” was discovered to have an inactivating mutation of the* Ob* gene in both alleles, causing a complete deficiency of the ob gene product, to date known as Leptin (Lep) [66]. Interestingly, overweight in these mice, with metabolic, endocrine, and immune disturbances, regressed under exogenous leptin administration [67].

Leptin, encoded by human* LEP* gene, on chromosome 7q31.3, is a 167-amino acid peptide with a four-helix bundle motif similar to that of a cytokine. It is produced, in a pulsatile manner, following a circadian rhythm, principally in adipose tissue but also in other different districts such as placenta, ovaries, and mammary epithelium [67]. Leptin and its receptor (LEPR) are present also in human spermatozoa and seminiferous tubules [68]. A few studies described human families with congenital leptin deficiency with early onset of obesity, hyperphagia, hypogonadotropic hypogonadism, and delayed puberty. Interestingly, inactivating mutation of the leptin receptor gene, encoded by human* LEPR* on chromosome 1p31, caused less severe clinical features, indicating that probably, in case of receptor dysfunction, leptin is able to interact with other molecules to exert its action [67, 68]. On the basis of recent findings, human Leptin is supposed to be implicated in the secretion of GnRH through stimulation of several hypothalamic neurons, secreting neuropeptide Y, proopiomelanocortin, and kisspeptin 1 [67, 68]. In the presence of leptin resistance, due to obesity, leptin is supposed to be unable to stimulate GnRH secretion, with consequent low levels of FSH and LH and hypogonadism [67, 68].

### 3.8. *TAC3 *and* TACR3*

Mammalian tachykinins comprise a protein family including substance P (SP), neurokinin A (NKA), neurokinin B (NKB), and hemokinin-1 (HK-1). Human tachykinins, characterized by a common C-terminal amino-acid sequence (Phe-X-Gly-Leu-Met-NH2) [69], are encoded by three different genes.* TAC1*,* TAC3,* and* TAC4* [70]. The* TAC3* gene encodes the NKB. Studies suggest that these peptides have a role as mediators of nonadrenergic and noncholinergic excitatory neurotransmission and recent data show that tachykinins are present in human spermatozoa and participate in the regulation of sperm motility [69]. Tachykinins (SP, NKA, and NKB) interact with three receptors, NK1R, NK2R, and NK3R [69, 71, 72]. The specific interactions of the three tachykinins with each of these receptors and their affinity vary as follows: SP > NKA > NKB for NK1R; NKA > NKB > SP for NK2R; NKB > NKA > SP for NK3R [71].

The particular ligand/receptor system NKB/NK3R, (encoded by* TAC3*/*TACR3*), previously investigated only for preeclampsia, alcohol, and cocaine dependence [73–75], gained an increasingly important role in human reproductive axis and in CHH onset in the last seven years. So far, over 40 CHH patients with* TAC3* and* TACR3* mutations have been reported, with a worldwide distribution [45, 76–79]. These patients are characterized by absence of pubertal development with low circulating levels of serum LH, low gonadal steroids and high prevalence of microphallus, indicating that NKB/NK3R signalling is essential for the normal activation of the reproductive axis late in gestation [45, 76–79]. Recent evidences suggest that NKB is able to modulate gonadotropin release through its action on Kiss1 neurons [71, 80], but many aspects of the physiology of the NKB/NK3R system in the context of reproduction remain to be fully characterized.

The* TAC3* gene maps on chromosome 12q13-q21 and it is composed of 7 exons, 5 of which are translated to form the preprotachykinin B peptide. This prepropeptide undergoes enzymatic cleavages to form first proneurokinin B, and then mature NKB. The amino acid sequence of the final active peptide is encoded by exon 5 only [81]. So far, three causative mutations, a splicing variant (c.209-1G>C) [12, 79], a frame-shift variant (G20fsX39) [76] and a missense variant (M90T) [78], were detected in CHH patients (Table 2). All these mutations (Table 2) occurred in homozygous state, suggesting an autosomic recessive inheritance.

The gene encoding the NKB receptor (*TACR3*), on chromosome 4q25, is composed by five exons. The identified mutations, either synonymous, or nonsynonymous or affecting physiologic splicing, are widely distributed along the gene, covering all main domains [12, 15, 76–78, 82] (Table 2).

### 3.9. *KISS1 *and* KISS1R (GPR54)*

Kisspeptin-1, encoded by* KISS1* gene, on chromosome 1q32, was early identified in 1996 as a suppressor of metastasis in human malignant melanoma [83]. The* KISS1* gene, encodes for a 54-amino acid peptide, also called kisspeptin 54, which corresponds to residues 68–121 of the preproprotein.

Its receptor, KISS1R, also known as GPR54, is encoded by a gene mapping on chromosome 19p13.3. In 2009, inactivating mutations in homozygous state in* KISS1R* were found in members of consanguineous families with a history of normosmic HH, revealing the reproductive roles of KISS1R and its ligand [11]. So far, mutations in the genes encoding the kisspeptin 1 and TAC3, as well as mutations in their receptors (KISS1R and TACR3, resp.), are associated with GnRH deficiency and a failure to initiate and/or progress through puberty [84]. Inactivating mutations in* KISS1* and* KISS1R* show an autosomal recessive pattern of transmission. Only few patients with* KISS1R* mutations have been reported to date [85]. Thanks to experiments in mice, researchers hypothesized that the system ligand/receptor TAC3/TACR3 is able to regulate the kisspeptin-1 release, which, through the interaction with the receptor KiSS-1R, stimulates the GnRH release and the normal reproductive function [80, 84].

### 3.10. *PCSK1*

Proprotein/neuroendocrine convertase deficiency, caused by rare mutations in* PCSK1* gene, has been associated with obesity, severe malabsorptive diarrhea, and certain endocrine abnormalities [86]. Neuroendocrine convertases are enzymes processing large precursor peptides to release bioactive fragments, as in the case of the proopiomelanocortin, which is processed by neuroendocrine convertase 1 (NEC 1), encoded by* PCSK1* gene, on chromosome 5q15-q21, in the corticotroph to produce adrenocorticotropic hormone and lipotropin [11]. The first* PCSK1* mutation, a compound heterozygous mutation, was identified in 1997, in a patient with obesity and hypogonadotropic hypogonadism (Gly483Arg and a donor splice site mutation in intron 5, causing skipping of exon 5 and the creation of a premature stop codon) [87]. In other two patients with a similar phenotype, a compound heterozygous mutation (Glu250X and Ala213del) and a homozygous Ser307Leu substitution were identified in* PCSK1* [88, 89]. So far,* PCSK1* is thought to act on GnRH prohormone processing, even if the molecular mechanisms are still unclear [11, 86].

### 3.11. *WDR11*

The* WDR11* gene, on chromosome 10q26, encodes for a 1224 amino acid protein, originally identified as a potential tumor suppressor in human glioblastoma cells [90]. The identification of human* WDR11* mutations in normosmic HH/KS, absent in controls, indicate that WDR11 plays an important role in human puberty. So far, five missense mutations were identified (R395W, H690Q, F1150L, A435T, and R448Q) [35, 91]. Four of these, R395W, H690Q, F1150L, and A435T are completely conserved in all 11 available mammalian orthologs, suggesting that these substitutions in six independent, sporadic patients could be very deleterious. All five described variants were in heterozygous state, suggesting an autosomal-dominant inheritance. The absence of truncating nonsense and frameshift mutations could indicate a more severe phenotype or an embryonic lethality [35, 91].

### 3.12. *HS6ST1*

Hs6st1 (Heparan sulphate 6-O-sulfotransferase 1), belonging to a class of molecules involved in neuronal development, is highly expressed in the brain [92]. Recently, the gene* HS6ST1*, mapping on chromosome 2q21, was found to be mutated in seven patients with hypogonadism with either normal olfaction (nHH) or variable degrees of olfactory dysfunction (KS) [92]. All identified mutations affect amino acid residues that are highly conserved in HS6ST1 but segregate as a complex trait in families, not following Mendelian criteria (Table 1). The study of Tornberg et al. [92] suggests that the identified* HS6ST1* missense mutations could not be sufficient to cause disease, indicating a probable cooccurrence of other mutated genes. Considering the importance of heparan sulfate in axon guidance during the brain development in mice [93], HS6ST1 is suggested to be important during the development and migration of GnRH neurons in humans.

### 3.13. *SEMA3A *and* SEMA7A*

It is well known that GnRH neurons are generated outside the brain, in the nasal placode, and migrate along olfactory/vomeronasal nerves reaching the hypothalamus by the time of birth. This migration occurs thanks to specific key players as semaphorins [94]. Their important role was reinforced with the identification of semaphorin mutations in patients with developmental neuroendocrine deficiencies associated with infertility [2, 94–96]. Some studies reported that a particular semaphorin, semaphorin 3A, encoded by human* SEMA3A*, on chromosome 7p12.1, if mutated both in humans and mice, could lead to abnormal migration of GnRH neurons to the hypothalamus, leading to hypogonadism and infertility [94–96] (Figure 1). So far, twelve different* SEMA3A* mutations, leading to KS phenotype, were detected in patients of both sexes [95–97]. All these variants were identified in heterozygous state, suggesting an autosomal dominant transmission, similar to the inheritance mode reported in KS patients with* FGFR1* mutations [39].

In 2011, a new candidate gene,* SEMA7A*, was added to the already long list of genes related to hypogonadotropic hypogonadism (Table 1) [98]. In this study* SEMA7A* was implicated in the normal development of the GnRH1 system in mice and was suggested to be a strong genetic marker for some forms of GnRH1 deficiency in humans. For the first time, in a recent study, mutations of the gene* SEMA7A*, mapping on human chromosome 15q22.3-q23, were detected in two hypogonadic patients, a male with nHH, harbouring also a* KISS1* mutation, and a male with KS, harbouring also a* KAL1* mutation [97]. On the basis of these results, Känsäkoski and colleagues suggest that the identified mutations are not sufficient, alone, to cause the pathology. Therefore, a di/oligogenic inheritance is assumed [97].

### 3.14. *LHB* and* FSHB*

Gonadotropins are eterodimers composed by a *α*-subunit, (common for TSH, FSH, LH, and hCG) and a specific *β*-subunit. So far, no mutations in the gene* CGA*, encoding the *α*-subunit, were identified, whereas, in some male and female patients, presenting with delayed puberty, several mutations were detected in the genes encoding the *β*-subunits of LH and FSH [99, 100].

Homozygous mutations in the* LHB* gene, (chromosome 19q13.32), abolishing the activity of LH, have been reported to date in seven men and two women [101–104]. In affected men, sexual differentiation is normal, but the absence of LH alters Leydig cells' proliferation and maturation, impairing spermatogenesis [101]. Women, harbouring an inactivating* LHB* mutation, have normal pubertal development and menarche, followed by oligomenorrhea and secondary amenorrhea [101]. Few data present in the literature, describing* LHB* mutations, suggest that one copy of the LH beta is sufficient for normal LH secretion and function of the gonadotropic axis, indeed, only the patients harbouring homozygous mutations showed hypogonadism whereas their relatives, harbouring a heterozygous variant, did not show clinical manifestations [101, 102].

The* FSHB* gene, located on chromosome 11p13, is composed of three exons but only exons 2 and 3 encode for the mature peptide. To date, four distinct* FSHB* mutations were described in four unrelated female patients with hypogonadism and three mutations were described in three CHH male patients [99, 100]. Affected women presented delayed puberty, lack of or poor breast development, and primary amenorrhea. After treatment with exogenous FSH, follicular maturation, ovulation, and fertility were achieved in two women. All affected men presented with small testes and azoospermia, but only one man presented absence of pubertal development [99, 100]. Data in the literature suggest that the presence of undetectable serum FSH and high serum LH levels in CHH patients of both sexes could be strongly due to molecular defects in the* FSHB* gene [99, 100].

### 3.15. *NDN*

Necdin belongs to the protein superfamily MAGE and it is able to activate GnRH expression and GnRH neurons development in rodents [105]. Human necdin, encoded by* NDN* gene (chromosome 15), has a potential role in the onset of hypogonadism in patients affected by Prader-Willi syndrome [106]. Few years ago, Beneduzzi and colleagues [105] identified a rare necdin variant in association with a mutation in* FGFR1*, in a patient with familial KS. Nevertheless, functional studies showed that the mutated necdin was able to activate the GnRH expression as the wild type protein [105]. Further studies are needed to clarify the role of this protein in puberty and in human reproduction.

### 3.16. *TSHZ1*

As mentioned before, the CHH subjects may show developmental abnormalities such as cleft palate, hearing loss, and other midline defects. However, it is unclear which CHH-associated gene is involved and to which extent in determining these developmental defects.

In 2007, the inactivation of the murine gene* Tshz1* demonstrated its role in the development of soft palate, axial skeleton, and middle ear in mice, suggesting the involvement of the human gene* TSHZ1* (Teashirt Zinc Finger Homeobox 1) in subjects with palate, skeletal, and ear abnormalities [107]. Few years later, investigating on small pool of individuals affected by syndromic orofacial cleft (OFC), palate abnormalities, and congenital aural atresia (CAA), mutations on* TSHZ1* gene were detected, showing an autosomal-dominant segregation [108, 109].

More recently, Ragancokova and colleagues investigated the role of* Tshz1* in mice, showing that the inactivation of this gene can cause olfactory bulb hypoplasia and a severe olfactory deficit [110]. In addition, this study evaluated olfaction of patients with* TSHZ1* heterozygous mutations, affected by CAA, and these showed hyposmia [110]. Further gene expression analyses showed a key role of* TSHZ1* in regulating the expression of* PROKR2*, which is associated to Kallmann Syndrome [110].

Considering these data, the* TSHZ1* gene is suspected to be involved, in a direct or indirect manner, and might be included in the panel of CHH-associated genes (Table 1).

## 4. Digenic and Oligogenic Inheritance

For a long period CHH were considered a monogenic pathology with Mendelian inheritance. In 2006, researchers began thinking about hypogonadotropic hypogonadism as a digenic disorder. Since that time, other researchers described cases of digenic mutations in normosmic HH or KS [14, 52]. Indeed, defects in different genes could act synergistically to induce the CHH/KS phenotype or to modify the severity of the GnRH deficiency [15, 52]. In 2010, Sykiotis et al. [14] identified 10 CHH patients harbouring rare digenic protein-altering variants and 18 CHH patients harbouring oligogenic known or predicted deleterious mutations. In 2011, Quaynor et al. [52] contributed to these data describing 48 normosmic CHH patients, screened for 13 disease-related genes. In 12.5% of these affected subjects digenic mutations were identified. Moreover, Quaynor and colleagues [52] suggested that a proportion of isolated GnRH deficiency could be attributable, in addition to digenic/oligogenic component, to nongenetic components, as in the cases of occasional adult onset of the disease after normal puberty and reproductive function in subjects without mutations.

Nevertheless, findings from the literature indicate that monogenic mutations account for most cases of CHH (over 80%) [14, 15, 52].

## 5. Conclusions

In conclusion, genetic alterations in twenty-four different genes, described so far in the literature and related to Central Hypogonadotropic Hypogonadism, have been reported. All these causative variants account for only 40–45% of affected patients, suggesting the involvement of other loci and/or epigenetic mechanisms. From the published data, the oligogenic nature of the disease emerges even more.

Considering the complexity of CHH, we believe that the best genetic investigative approach could be the use of laboratory methods, such as next generation sequencing (NGS), allowing the simultaneous screening of many genes.

To date, little attention was given to the many nonpathogenic single nucleotide polymorphisms (SNPs) found in these genes. Although the polymorphic variants, described in the literature, are not considered pathologic, they could influence the phenotype, especially if occurring in combination with other mutations in other genes. Therefore, we believe that their role should not be underestimated and future studies correlating polymorphisms, causative mutations (if any), and clinical characteristics of patients should be conducted.

## Figures and Tables

**Table 1:** Human genes involved in CHH.

Genes	Location	Gene ID	Structure (coding exons)	Inheritance	OMIM	Phenotype
*KAL1 *	Xp22.3	3730	14	XR	308700	KS
*FGFR1 (KAL2) *	8p12	2260	17	AD	147950	KS
*FGF8 (KAL6) *	10q24	2253	6	AD	612702	nHH
*PROK2 (KAL4) *	3p13	60675	4	AR	610628	KS
*PROKR2 (KAL3) *	20p12.3	128674	2	AD, AR	147950	KS, nHH
*CHD7 (KAL5) *	8q12.2	55636	37	AD	612370	CHARGE, KS, nHH
*NELF *	9q34.3	26012	14	Digenic	614838	KS
*GNRH1 *	8p21-p11.2	2796	3	AR	614841	nHH
*GNRHR *	4q21.2	2798	3	AR	146110	nHH
*LEP *	7q31.3	3952	2	AR	614962	nHH
*LEPR *	1p31	3953	18	AR	614963	nHH
*TAC3 *	12q13-q21	6866	5	AR	614839	nHH
*TACR3 *	4q25	6870	5	AR	614840	nHH
*KISS1 *	1q32	3814	2	AR	614842	nHH
*KISS1R (GPR54) *	19p13.3	84634	5	AR	614837	nHH
*PCSK1 *	5q15-q21	5122	14	AR	162150	nHH
*WDR11 *	10q26	55717	29	AD	614858	KS, nHH
*HS6ST1 *	2q21	9394	2	Unclear	614880	KS, nHH
*SEMA3A *	7p12.1	10371	17	AD, di/oligogenic	614897	KS
*SEMA7A *	15q22.3-q23	8482	14	Di/oligogenic	607961	nHH, KS
*LHB *	19q13.32	3972	3	AR	152780	nHH
*FSHB *	11p13	2488	2	AR	136530	nHH
*NDN *	15q11.2-q12	4692	1	Unknown	602117	KS, Prader-Willi
*TSHZ1 (candidate) *	18q22.3	10194	1	AD	614427	Expected: OFC, CAA, hyposmia, probably KS

Gene ID: identification number assigned to a specific gene in NCBI database; OMIM: online catalogue of human genes and genetic disorders, numbers refer to the first search result including the gene name and the term “hypogonadism;” KS: Kallmann syndrome; nHH: normosmic hypogonadotropic hypogonadism; XR: X-linked recessive; AR: autosomic recessive; AD: autosomic dominant; OFC: syndromic orofacial cleft; CAA: congenital aural atresia.

**Table 2:** Nucleotide variants identified so far in the two human genes *TAC3* and *TACR3*.

Gene	Variants		
	Synonymous	Non synonymous	Splicing
*TAC3 *		G20fsX39 M90T	c.209-1G>C

*TACR3 *	L58L V98V T246T S448S	G18D S27X G93D Y145X H148L W208X I249V Y256H W275X R295S Y315C	IVS1+1delG c.738-1G>A
